# The Enigmatic Interplay of Interleukin-10 in the Synergy of HIV Infection Comorbid with Preeclampsia

**DOI:** 10.3390/ijms25179434

**Published:** 2024-08-30

**Authors:** Shirelle Janine Naidoo, Thajasvarie Naicker

**Affiliations:** Department of Optics and Imaging, Nelson R. Mandela School of Medicine, University of KwaZulu-Natal, Durban 4001, South Africa; naidoos15@ukzn.ac.za

**Keywords:** interleukin-10, preeclampsia, human immunodeficiency virus

## Abstract

Cytokines coordinate the intricate choreography of the immune system, directing cellular activities that mediate inflammation, pathogen defense, pathology and tissue repair. Within this spectrum, the anti-inflammatory prowess of interleukin-10 (IL-10) predominates in immune homeostasis. In normal pregnancy, the dynamic shift of IL-10 across trimesters maintains maternal immune tolerance ensuring fetal development and pregnancy success. Unravelling the dysregulation of IL-10 in pregnancy complications is vital, particularly in the heightened inflammatory condition of preeclampsia. Of note, a reduction in IL-10 levels contributes to endothelial dysfunction. In human immunodeficiency virus (HIV) infection, a complex interplay of IL-10 occurs, displaying a paradoxical paradigm of being immune-protective yet aiding viral persistence. Genetic variations in the IL-10 gene further modulate susceptibility to HIV infection and preeclampsia, albeit with nuanced effects across populations. This review outlines the conceptual framework underlying the role of IL-10 in the duality of normal pregnancy and preeclampsia together with HIV infection, thus highlighting its regulatory mechanisms and genetic influences. Synthesizing these findings in immune modulation presents avenues for therapeutic interventions in pregnancy complications comorbid with HIV infection.

## 1. Introduction

In the delicate symphony of the human immune system, cytokines act as the conductor, directing and modulating cellular activities for the orchestration of inflammation, defense against pathogens, tissue repair, and the maintenance of homeostasis [1]. More specifically, these small pleiotropic proteins serve as messengers, transmitting signals between cells to regulate immunity, inflammation and hematopoiesis [2,3]. Cytokines exhibit an extensive range of functions, operating in autocrine, paracrine and endocrine manners to exert their effects on nearby or distant cells [4]. Their functions span across different stages of the immune response, from the initial detection of threat to the combating of the pathogen and tissue repair [5].

At the onset of foreign threat detection, cytokines serve to alert the immune system [6]. Pro-inflammatory cytokines such as Interleukin-1 (IL-1), tumor necrosis factor-alpha (TNF-α), and Interleukin-6 (IL-6) are released in response to pathogens, trauma, or stress. These molecules initiate and amplify the immune response by recruiting immune cells to the site of infection or injury and stimulating their activation and proliferation [7]. Once immune cells are recruited, cytokines modulate their functions [8]. Interferons, for example, help combat viral infections by interfering with viral replication and enhancing the activity of natural killer (NK) cells and macrophages [9]. Interleukins such as Interleukin-2 (IL-2) are critical for T-cell proliferation and activation which are essential for adaptive immune responses [10].

Moreover, cytokines are essential for maintaining the proper homeostatic balance between various immune responses. For example, under the influence of specific cytokines, T helper (Th) cells can differentiate into distinct subsets (i.e., Th1, Th2, Th17, Treg) [11]. The different cytokines produced by these subsets of cells lead to mutually exclusive functional properties. Of note, Th1 cells activate macrophages and mediate immune responses against intracellular pathogens, while Th2 cells stimulate B cells [11].

Cytokines have a dual function in immune regulation; in addition to immune activation, they also regulate immune response resolution [4]. Anti-inflammatory cytokines, including Interleukin-10 (IL-10) and transforming growth factor-beta (TGF-β), act as brakes, dampening excessive inflammation and preventing collateral tissue damage [12]. They aid in restoring homeostasis by promoting tissue repair and re-instating the immune response to a basal state [13].

In addition, cytokines influence various physiological processes such as embryonic development and even neuroendocrine function [14,15]. Cytokines like erythropoietin (EPO) and thrombopoietin (TPO) regulate red blood cell and platelet production, respectively, demonstrating their involvement in hematopoiesis [16]. Although cytokines are indispensable for maintaining health, a dysregulation or imbalance in cytokine production may lead to pathological conditions [17]. Excessive or prolonged inflammation, often termed “cytokine storms”, can contribute to autoimmune diseases, allergies, and severe conditions such as septic shock and SARS-CoV-2 infection [18]. Therefore, understanding the multifaceted functions of cytokines is paramount and will not only elucidate immune system complexities but may also unveil potential therapeutic targets for diseases driven by immune dysregulation.

## 2. Interleukin-10

Amidst the myriads of cytokines orchestrating immune responses, IL-10 stands out as a key regulator, revered for its potent anti-inflammatory properties and its crucial role in maintaining immune homeostasis [19]. Initially discovered for its ability to inhibit cytokine synthesis by Th1 cells, the IL-10 gene is located on chromosome 1 at position 1q31-32 and is approximately 4.7 kb in length, containing four introns and five exons [20]. Its core function lies in its ability to downregulate pro-inflammatory cytokine production via the inhibition of cytokines such as interferon-gamma (IFN-γ), IL-2, and TNF-α, which are known drivers of inflammation [21]. By dampening the production of these inflammatory mediators, IL-10 functions as a robust inhibitor, curbing excessive immune response and preventing tissue damage that may result from uncontrolled inflammation. In therapeutic settings, approaches to boost IL-10 production or deliver it to inflamed areas are being harnessed as prospective therapeutic modalities for mitigating inflammatory disorders [22]. However, the complex interactions within the immune system pose challenges to precisely modulating the IL-10 response without disrupting overall immune function.

## 3. The Role of IL-10 in Normal Pregnancy

Interleukin-10 is pivotal in mediating the delicate homeostatic equilibrium between pro-inflammatory and anti-inflammatory signals required to ensure a successful pregnancy outcome [23]. During pregnancy, placental trophoblasts and Tregs are the main producers of IL-10, which binds to its cognate receptor (IL-10R), a heterodimer made up of IL-10R1 and IL-10R2 subunits and affects macrophages and DCs. IL-10R1 induces a conformational shift in IL-10 by binding to IL-10 specifically, allowing oligomerization with IL-10R2 [24]. This triggers the transcription of anti-inflammatory genes by activating the Janus Kinase-Signal Transducer of Activation (JAK-STAT) signaling pathway, especially STAT3 [25]. As a result, pro-inflammatory cytokines including TNF-α, IL-6, and IL-12 are suppressed, limiting the risk of severe inflammatory reactions that could endanger the growing fetus [24]. IL-10 contributes significantly to immune tolerance and fetal development via a multifaceted role across the trimesters of pregnancy [26]. The transposition of IL-10 across trimesters establishes a conducive environment for successful gestation and fetal growth as it manages maternal inflammation and cytokine crosstalk at the maternal–fetal junction [27]. Studies indicate a surge in IL-10 production during early and middle gestation; however, these levels appear to decrease at term, with a subsequent elevation post-delivery [28]. Within the human placenta, IL-10 expression is localized to the invasive cytotrophoblasts, which are speculated to prompt the expression of human leukocyte antigen G (HLA-G), thereby aiding in immune regulation [29]. Early investigations of IL-10 in human pregnancy highlight its role in modulating placentation by inhibiting matrix metalloproteinase 9 (MMP-9), an enzyme involved in degrading the extracellular matrix to facilitate cytotrophoblast migration from the decidua into the myometrium in a set timed sequence [29]. Early in the first trimester, trophoblasts demonstrate a temporary reduction in IL-10 production, concomitant with an elevation in MMP-9 levels, indicating a deliberate downregulation of IL-10 to facilitate their invasive behavior [23]. Moreover, these studies firmly position IL-10 as a crucial participant in the dynamic shift of vascular remodeling, also referred to as the physiological conversion of spiral arteries required for a successful pregnancy.

## 4. First Trimester

In healthy pregnancies, the first trimester is characterized by blastocyst implantation and initial placentation, marking the onset of a critical phase essential for establishing pregnancy [30]. This initial stage of pregnancy is characterized by a predominance of pro-inflammatory changes and increased IL-10 production [31]. During implantation, the blastocyst cleaves the epithelial lining of the uterus with resultant endometrial tissue degeneration. This is followed by remodeling of the maternal blood vessels involving replacement of the endothelium and vascular smooth muscle with a fibrinoid type material in which trophoblastic cells are embedded [32]. This vascular adaptation ensures optimal blood flow to support nutrient, and oxygen demands by the growing fetus. To facilitate repair of the uterine epithelium, localized activation of inflammatory mediators occurs [33]. This inflammatory response together with the associated hormonal changes and increased gonadotropins manifests clinically in the mother as morning sickness [34]. During this time, IL-10 serves as a key player in fostering maternal immune tolerance towards the fetal semi-allograft [35]. It contributes to the suppression of immune responses that may otherwise target the developing embryo [36]. The early embryo itself secretes IL-10, which aids in regulating the immune milieu at the maternal–fetal interface. This localized production of IL-10 contributes to the maintenance of a tolerogenic environment, preventing maternal immune rejection of the embryo [26].

## 5. Second Trimester

During the second trimester, the symptoms of morning sickness tend to subside, attributed largely to the predominant anti-inflammatory phase that is characterized by increased Th2 cytokine production and increased IL-10 levels [37]. The role of IL-10 remains pivotal in maintaining immune tolerance and supporting fetal development, with the placenta becoming the predominant source of IL-10 production [38]. The placenta serves as a barrier between the maternal and fetal compartments, secreting IL-10, which effectively attenuates excessive inflammation and prevents immune-mediated damage to the developing fetus [28]. Moreover, the anti-inflammatory properties of IL-10 help in regulating the maternal immune response to fetal antigens, ensuring that the immune system does not mount an adverse reaction against the growing fetus. During this phase, the fetus undergoes rapid growth and development. The mother, placenta, and fetus are now well adapted to one another and have established a symbiotic relationship driven by the shift of cytokine balance towards the anti-inflammatory Th2 pathway [39,40]. 

## 6. Third Trimester

The last phase of pregnancy is parturition, which prompts the re-instatement of the pro-inflammatory milieu [27]. During this final stage of pregnancy, elevated levels of IL-10 are observed in serum and amniotic fluid and are critical for maintaining pregnancy during this trimester [36]. Parturition is initiated by an increase of pro-inflammatory cytokines within the amniotic fluid coupled with an influx of immune cells which invade the fetal membranes, cervix, and myometrium [32]. This invasion facilitates the contraction of the uterus to aid in delivery of the fetus and rejection of the placenta. A premature decline in IL-10 levels may be associated with pre-term birth [36]. The regulation of inflammation is therefore essential to a healthy pregnancy, and interruptions or changes to maternal inflammatory response are associated with several adverse pregnancy outcomes [27]. Understanding the conceptual framework underlying the paradoxical shift of IL-10 across pregnancy trimesters is required to better understand its dynamic function and to comprehend the complexities of maternal–fetal immune interactions. Furthermore, it offers insight into potential strategies for managing pregnancy-related complications.

## 7. Preeclampsia

Preeclampsia is one such pregnancy-associated malady characterized by the onset of hypertension, and/or proteinuria, and/or end-organ dysfunction [41]. Preeclampsia is one of the most common direct causes of maternal and perinatal mortality and morbidity. It affects 2–8% of pregnancies globally [42]. Although clinical symptoms usually present at 20 weeks of gestation, the initiating events of disease are hypothesized to begin as early as implantation [43]. The precise mechanisms underlying preeclampsia are complex and multifactorial, with emerging evidence suggesting a dysregulated immune response, involving altered cytokine production and subsequently weakened immunological tolerance [44,45].

In normal pregnancy, the decidua, which is composed of immune cells such as macrophages, uterine natural killer (NK) cells, dendritic cells (DCs), and regulatory T cells (Tregs), establishes the placenta through a process known as decidualization [46]. During decidualization, fetal trophoblast cells invade the decidua and migrate into the myometrium. The extracellular matrix and endometrial vessels undergo remodeling resulting in high-capacity, low-resistance vasculature capable of supplying increased blood flow to the placenta and fetus [43]. During preeclampsia however, the imbalance between pro- and anti-inflammatory cytokines, including diminishing IL-10 levels, preempts an inflammatory state that promotes oxidative stress, anti-angiogenic milieu, and endothelial dysfunction [47]. These factors contribute to the systemic vascular abnormalities observed in preeclampsia, leading to the characteristic symptoms of hypertension, proteinuria, and impaired blood flow to vital organs [48]. 

Uterine DCs promote an anti-inflammatory dominant state in the uterus and are critical for both endometrial decidualization and maternal immunotolerance of the fetus [49]. In addition, inflammatory DCs and cytotoxic cells decrease in circulation coupled with the hormonal downregulation of B-cell differentiation and placental reactive B-cells. These changes are strictly regulated by the release of cytokines and anti-angiogenic factors and inhibit reactivity to the fetus and placenta [43].

**Figure 1:**
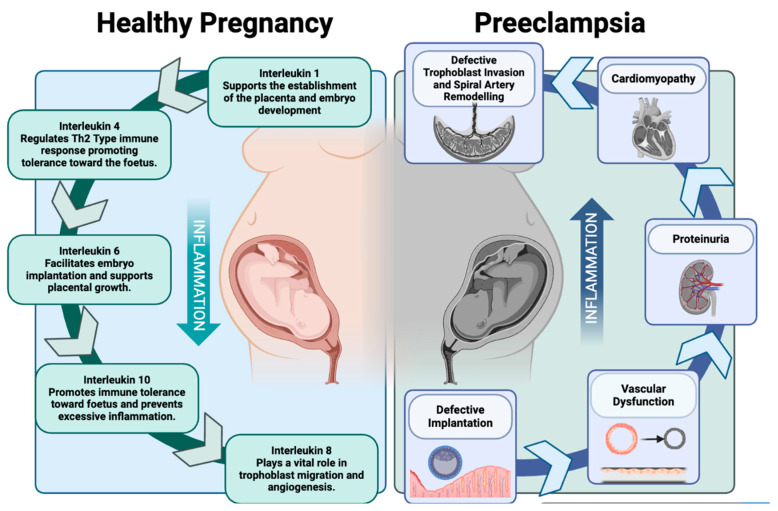
Healthy pregnancy versus preeclampsia. This image depicts the regulation of inflammation during a healthy pregnancy in comparison to the dysregulation observed during preeclampsia and the associated outcomes of this dysregulation (Created using BioRender.com).

In the context of preeclampsia, studies have indicated aberrant levels of IL-10 in women with preeclampsia compared to healthy pregnancies [50]. In addition, a study conducted by Ferguson et al. explored the longitudinal trajectories of a panel of inflammatory markers, including IL-10, in women who developed preeclampsia as well as normotensive pregnancies and demonstrated that inflammatory markers exhibit varying patterns across pregnancy beginning as early as 10 weeks in preeclamptic pregnancies compared to normotensive pregnancies [51]. IL-10, known for its anti-inflammatory properties, plays a crucial role in maintaining immune tolerance by preventing excessive inflammation thus resulting in the immunosuppressed state observed in normal pregnancy [52]. Preeclampsia is characterized by chronic inflammation and endothelial damage attributed to impaired IL-10 signal transduction, thus interrupting the JAK-STAT pathway resulting in downstream IL-10 downregulation [53]. Rather than promoting immunotolerance alone, immune cells such as CD4+ Th1 cells, cytotoxic NK cells, and autoreactive B cells secrete factors that instigate an increase in innate immune activation and inflammation resulting in defective extravillous trophoblast invasion and impaired remodeling of the spiral arteries [54]. Insufficient blood, oxygen, and nutrients are delivered to the fetus, resulting in placental ischemia and augmented oxidative stress. The hypoxic placenta in turn secretes elevated levels of anti-angiogenic factors (soluble fms-like tyrosine kinase-1 and sEnd) that promote vasoconstriction and increase maternal blood flow in an attempt to increase the supply of oxygenated maternal blood to the fetus [55]. This shift in immune response illustrates the role of chronic inflammation in the development and progression of preeclampsia (Figure 1) [43]. Strategies aimed at restoring immune balance and targeting IL-10 pathways to mitigate excessive inflammation and promote immune tolerance could hold promise for therapeutic interventions or the prevention of preeclampsia. However, the complexities of immune regulation during pregnancy pose a challenge in developing targeted therapies without compromising the delicate balance required for maternal–fetal tolerance. Further research into the immune mechanisms and the role of IL-10 in pregnancy complications is therefore crucial.

## 8. The Influence of IL-10 Promoter Region SNPs on Preeclampsia

The promoter region of IL-10 contains at least 27 polymorphic sites which alter gene transcription and expression and consequently IL-10 secretion [56]. These polymorphisms result in aberrant cytokine production which influence disease susceptibility and the severity of several inflammatory conditions including preeclampsia [57]. Several studies have since investigated variants within the IL-10 promotor region in an effort to elucidate their effect on disease susceptibility based on ethnicity and severity of condition. A study conducted by Sowmya et al. (2014) explored the relevance of IL-10 promoter polymorphisms in early onset preeclampsia (EOPE) amongst individuals of Indian ancestry, specifically −1082G>A, −819C>T, and −592C>A. The authors reported an increased incidence of EOPE in patients expressing the C allele of −819C>T or the A allele of −592C>A but found no association between EOPE and −1082G>A of the IL-10 gene promoter region [58]. One year later, Song and Zhong (2015) performed a comparable study involving individuals of Chinese descent. Their findings supported the absence of a link between −1082G>A and −819C>T with EOPE development. The authors did, however, find a notably higher occurrence of EOPE in patients with the CC and AC + CC genotypes of −592C>A. Nonetheless, they advised exercising caution in interpreting their results, citing the limited sample size and the potential for selection bias [59]. In the same year, another study conducted on a Chinese population assessed the influence of −819C>T and −1082G>A on the risk of preeclampsia development and reported an absence of a significant association between −1082G>A and the development of preeclampsia but did observe an elevated risk of preeclampsia development in individuals with the CC genotype of −819C>T [60]. Zubor et al. (2015) illustrated an association between the A allele of −592C>A and the development of severe preeclampsia in a Slovakian population stratified into normotensive pregnant, preeclamptic, and women diagnosed with hypertensive disorders of pregnancy (HDP) [61]. Conversely, a study involving Sinhalese women showed no significant association between IL-10 promoter SNPs, specifically −824C>T, −592C>A, and −1082G>A, and the risk of developing preeclampsia [62]. Interestingly, ongoing debate surrounds which −1082G>A allele (A/G) is linked to the development of preeclampsia in various other populations [63]. In Turkey, a study of the −1082G>A SNP revealed a three-fold increased risk of preeclampsia development in patients expressing the AA compared to the GG genotype [64]. Likewise, a study conducted in the Minas Gerais region of Brazil revealed a protective association between the G allele of −1082G>A and the development of preeclampsia by demonstrating a higher frequency of the G allele in normotensive pregnant compared to preeclamptic women. This study, however, consisted of 226 participants of which only 54 were preeclamptic compared to 172 that were normotensive [65]. Nonetheless, their finding is supported by a more recent study conducted in Uzbekistan, which consisted of 71 women at risk of developing preeclampsia and 50 already preeclamptic women. The authors reported a significantly increased likelihood of preeclampsia development in women expressing the A allele of −1082G>A, whilst those expressing the G allele were significantly less likely to develop preeclampsia [66]. In direct contrast, Zhou et al. (2018) reported a significantly increased risk of preeclampsia in women expressing the G allele within a Chinese population [63]. A study conducted in the North African country of Tunisia revealed a significant association between the T allele and T/T genotype of −819C>T and susceptibility to preeclampsia development. The authors further reported that the T variant and T/T genotype corresponded with decreased IL-10 production. They reported no significant association between the SNPs, −1082G>A and −592C>A and preeclampsia; however, they observed an increased incidence of preeclampsia development with concomitant decreased IL-10 production in women expressing the ATA haplotype of −1082G>A, −819C>T, and −592C>A [38]. An earlier analysis of IL-10 promotor haplotypes and their influence on susceptibility to preeclampsia reported no association between the ATA haplotype and risk of preeclampsia. The latter study, however, reported a two-fold increased risk of preeclampsia development in women expressing the ACC haplotype [67]. In contrast, a study conducted on Iranian women reported no significant association of preeclampsia with any of the three most common reported haplotypes (ATA/GCC/ACC) of the IL-10 promoter SNPs [68].

Despite numerous attempts to correlate the impact of IL-10 SNPs with increased susceptibility to preeclampsia development, results remain inconsistent due to variations in selection criteria, sample size, ethnic diversity, and linkage disequilibrium. To synthesize findings and direct future research, several reviews and meta-analyses have been undertaken. A systematic review and meta-analysis conducted by Nath et al. evaluated 56 studies and concluded that preeclamptic women exhibited decreased IL-10 levels compared to healthy controls [50]. Zhang et al. (2016) examined the association between chromosome 1 polymorphisms (−1082G>A and −824C>T) of the IL-10 gene and preeclampsia susceptibility and concluded that there was an absence of a significant link between these polymorphisms and preeclampsia risk [69]. A more recent review by Che et al. (2021) similarly found no significant association between susceptibility to preeclampsia and SNPs −819C>T and −592C>A across diverse ethnicities [70]. However, they did note a significant association in patients of Caucasian or Asian ethnicity. These disparities suggest that SNPs linked to disease susceptibility and severity may be ethnicity-specific, emphasizing the need for future studies to target overlooked populations. Confounding variables, which may not have been included in research but could potentially have an impact on IL-10 levels, should also be noted. According to a McLeod et al. study, pregnant women’s perceived stress is linked to a decrease in IL-10 production [71]. Furthermore, some metabolites have also been shown to have an impact on the production of IL-10, suggesting that diet may have a role in controlling the synthesis of IL-10 [36]. According to Steckle et al.‘s research on asymptomatic pregnant women who went into spontaneous preterm labor, low-intensity exercise also exhibits an indirect correlation with IL-10 production in pregnant women [72].

## 9. Human Immunodeficiency Virus

Human Immunodeficiency Virus (HIV) infection poses a significant global health challenge, ranking as the fourth leading cause of worldwide mortality [73]. The virus targets CD4+ T cells, leading to immunocompromise, initially infecting mucosal tissues before spreading to lymphoid organs and the bloodstream within days, with peak infectiousness occurring around 30 days post-infection. Subsequently, viral replication slows, allowing individuals to live with controlled infection for up to a decade without progressing to AIDS [74]. A prominent aspect of HIV infection involves the expression of multiple pro-inflammatory cytokines that contribute to immune suppression by engaging in T cell apoptosis. These cytokines play a critical role in the HIV life cycle, particularly in promoting the virus’s capacity to establish latent reservoirs at the transcriptional level [75]. Certain viral proteins necessary for disease progression have the capability to exploit pro-inflammatory cytokine signaling, underscoring the significance of inflammation in HIV’s pathogenesis. In vivo studies have revealed a direct correlation between chronic inflammation and heightened viremia along with hastened disease progression [76]. The persistent activation of the innate immune system is responsible for chronic inflammation in HIV infection and is directly linked to the rapid depletion of CD4+ T cells [77]. This depletion impairs their ability to mount effective antiviral responses while deteriorating the intestinal epithelium, facilitating increased circulation of bacterial products and prompting an observable immune response [78,79]. These insights have drawn attention to the pivotal role of cytokines in the chronic inflammation characteristic of HIV infection.

IL-10 in particular plays a complex role in the context of viral infections such as HIV. Its involvement in regulating immune responses during HIV infection has been a subject of intense study, revealing both beneficial and detrimental aspects of IL-10’s influence on viral pathogenesis and disease progression [80]. Initially hailed as a protective factor due to its ability to downregulate inflammatory responses, IL-10 was believed to mitigate immune activation and limit HIV-induced pathology [81]. Its anti-inflammatory actions were thought to control the hyperactivation of the immune system, contributing to HIV-associated immunopathogenesis [82]. However, subsequent research has recently unveiled a more paradoxical role for IL-10 in HIV infection. While it exerts anti-inflammatory effects, IL-10 has also been associated with immunosuppression, hampering effective antiviral immune responses [81]. IL-10 hinders the body’s ability to combat HIV by suppressing the activation and function of key immune cells like CD4+ T cells and antigen-presenting cells. This suppression weakens the immune system’s ability to detect and fight the virus effectively. Moreover, IL-10 encourages the production of regulatory T cells, which further dampen immune responses by inhibiting the activity of T cells responsible for targeting HIV. Additionally, IL-10 reduces the production of pro-inflammatory cytokines needed to coordinate an effective immune response against HIV. In essence, IL-10’s actions contribute to a less robust immune response, which enables HIV to evade detection and persist as latent reservoirs in the body. HIV infection triggers increased production of IL-10 by various immune cells, creating an environment conducive to viral persistence and immune evasion [83]. Moreover, IL-10’s impact extends beyond its direct effects on immune cells. It can modulate the activity of other cytokines and chemokines involved in the immune response against HIV [80]. For instance, IL-10 can suppress the production of interferons, which play a crucial role in inhibiting viral replication. This suppression further contributes to the establishment of a microenvironment that favors HIV amplification and persistence [84]. IL-10 has the potential to disrupt the delicate balance between immune activation and regulation. Comprehension of this balance is therefore critical for tailored treatment interventions aimed at modulating IL-10 levels or activity to restore immune balance without compromising antiviral responses. However, the dual nature of IL-10’s effects in HIV infection poses challenges in designing interventions that selectively target its beneficial aspects while mitigating its detrimental impact on antiviral immunity.

## 10. IL-10 Gene Polymorphisms and Their Role in HIV Susceptibility

**Figure 2:**
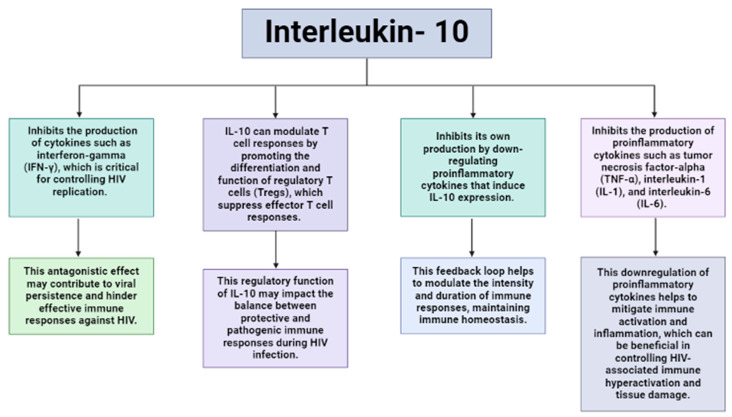
The role of interleukin-10 in Human Immunodefiency Virus (HIV) infection. This flow diagram depicts the double-edged role Interleukin-10’s anti-inflammatory properties play in HIV infection.

Owing to the clear relationship between IL-10 and HIV infection (Figure 2), the association between SNPs in IL-10 and HIV pathogenesis has garnered substantial attention [85]. Naicker et al. (2009) demonstrated that IL-10 gene SNPs linked to heightened IL-10 expression correlated with a reduced risk of HIV-1 infection; however, individuals with these SNPs were more prone to higher viral loads during the initial 3 months of infection. Interestingly, the authors observed a reversal of this association as disease progressed. The authors further demonstrated that among their South African cohort, those that expressed the AA genotype of −592 C>A were at higher risk of HIV infection [86]. This finding was supported by Shin et al. (2000) and Shrestha et al. (2010) who reported similar findings in their North American cohort and African American cohorts, respectively [85,87]. In contrast, Erikstrup et al. (2007) reported a protective role of the G allele of the IL-10 promotor polymorphism, −1082 G>A. These authors reported increased IL-10 production, attenuated by a CD4+ T cell decrease and improved survival in Zimbabwean carriers of this allele [88]. To date, a number of allelic variants in the HLA system, cellular immunity, chemokine receptors and ligands, and cytokines have demonstrated an observable effect on HIV infection, AIDS progression, or outcomes of disease [73]. 

## 11. Antiretroviral Therapy and Its Effect on Interleukin-10

Antiretroviral therapy (ART), also known in the contemporary literature as Highly Active Antiretroviral Therapy (HAART) or Combined Antiretroviral Therapy (cART), encompasses a range of pharmacological interventions aimed at combating HIV by targeting various stages of its life cycle [89]. Nucleoside Reverse Transcriptase Inhibitors (NRTIs) and Non-Nucleoside Reverse Transcriptase Inhibitors (NNRTIs) play a crucial role in hindering the reverse transcriptase enzyme through distinct binding sites, thereby interrupting the conversion of viral RNA into DNA. Examples of this drug class include Tenofovir, Emtricitabine, Efavirenz, and Rilpivirine as prominent examples [90]. Protease Inhibitors (PIs), such as atazanavir and darunavir, obstruct HIV’s maturation by inhibiting the protease enzyme which is indispensable for viral replication [91]. Integrase Strand Transfer Inhibitors (INSTIs), including Dolutegravir and Bictegravir, impede the integrase enzyme, thereby preventing the incorporation of viral DNA into the host genome [92]. Lastly, entry inhibitors, represented by enfuvirtide and maraviroc, prevent HIV’s entry into immune cells by hindering its capacity to bind to these cells [93]. These therapeutic agents collectively constitute the foundation of current ART regimens, providing a comprehensive strategy for managing HIV infection by addressing various phases of the virus’s replication cycle. Initial investigations into the impact of HAART on IL-10 disclosed a significant, albeit gradual, reduction in IL-10 levels, without normalization, highlighting IL-10’s pathogenic role in HIV infection [94]. This observation has been corroborated by later studies, which documented diminished IL-10 levels following ART intervention [81,95,96]. Further evidence of ART’s suppressive effect on IL-10 emerged from the observed increase in IL-10 levels upon ART interruption, leading to reduced IL-10 levels becoming an indicator of effective ART [97,98]. Notably, variations within the IL-10 gene pathway have also been implicated in influencing immunological response to HAART. While several of these studies have been conducted in European study populations, fewer investigations have sought to explore this effect in minority populations [85].

## 12. The Synergy of HIV-Infected Preeclamptic Women

As elucidated, IL-10 assumes a critical regulatory role in maintaining an anti-inflammatory milieu conducive to healthy pregnancy [99]. However, in the context of preeclampsia, this regulatory function is perturbed, leading to diminished IL-10 production and exacerbation of inflammatory processes [53]. Intriguingly, IL-10 demonstrates a positive correlation with HIV viral load, whereby heightened IL-10 levels correlate with increased viral replication and disease progression [29,94,100]. This juxtaposition presents a paradox in treatment-naïve HIV-infected women who develop preeclampsia. It would be anticipated to observe a less severe form, if any, of preeclampsia in individuals with elevated viral loads due to IL-10’s purported anti-inflammatory properties, which could mitigate the inflammatory milieu during gestation. Conversely, those with lower viral loads and no prior treatment might manifest more severe forms of preeclampsia. The introduction of antiretroviral therapy introduces further complexity to this scenario, as it has been posited to suppress IL-10 levels [96,101]. Consequently, we would hypothesize that individuals with low viral loads and severe preeclampsia could be observed in this context. While we speculate about these potential outcomes, it is imperative to conduct further investigations on this subset of patients to draw more definitive conclusions that can serve as a foundation for future research endeavors. A promising avenue for therapeutic intervention is the use of recombinant human IL-10. This method was tested in clinical trials for treating a plethora of inflammatory conditions including rheumatoid arthritis, inflammatory bowel disease, psoriasis, organ transplantation, and chronic hepatitis and could be adapted to preeclampsia [26]. Experimental studies performed by Cheng and Sharma, as well as others, have demonstrated that the administration of recombinant IL-10 reverses or alleviates symptoms of many adverse pregnancy outcomes in animal models, suggesting that the IL-10/IL-10 receptor (IL-10R) axis could potentially become a target for therapeutic intervention and treatment of adverse pregnancy outcomes [26].

## 13. Conclusions

This review elucidates the differential expression of IL-10 across the trimesters of normal gestation. In the context of preeclampsia, there is, however, a reduction in IL-10 production, which exacerbates inflammatory responses. Concurrently, the equilibrium of maternal immunity is altered during HIV infection, leading to increased IL-10 levels that facilitate viral propagation and dissemination. However, in the comorbid presence of HIV infection and preeclampsia, IL-10 levels are downregulated, attributable to the immune-restorative effects of ART. It is pertinent to note that both HIV infection and preeclampsia are implicated in endothelial dysfunction. Moreover, the endothelial dysfunction associated with preeclampsia may amplify HIV-related complications, including thrombosis and cardiovascular disease. Vascular alterations induced by preeclampsia may also aggravate fetal distress, thereby elevating the risk of vertical transmission of HIV. Although the pivotal role of IL-10 in both pathologies is acknowledged, its utility as a preventive or therapeutic agent, particularly in comorbid scenarios, remains to be fully elucidated. This interaction accentuates the need for integrated clinical management, especially in low- to middle-income regions such as South Africa. Future investigations should aim to delineate the relationship between IL-10 and the co-occurrence of HIV and preeclampsia, with the objective of uncovering novel therapeutic avenues.
